# Utility of Climatic Information via Combining Ability Models to Improve Genomic Prediction for Yield Within the Genomes to Fields Maize Project

**DOI:** 10.3389/fgene.2020.592769

**Published:** 2021-03-08

**Authors:** Diego Jarquin, Natalia de Leon, Cinta Romay, Martin Bohn, Edward S. Buckler, Ignacio Ciampitti, Jode Edwards, David Ertl, Sherry Flint-Garcia, Michael A. Gore, Christopher Graham, Candice N. Hirsch, James B. Holland, David Hooker, Shawn M. Kaeppler, Joseph Knoll, Elizabeth C. Lee, Carolyn J. Lawrence-Dill, Jonathan P. Lynch, Stephen P. Moose, Seth C. Murray, Rebecca Nelson, Torbert Rocheford, James C. Schnable, Patrick S. Schnable, Margaret Smith, Nathan Springer, Peter Thomison, Mitch Tuinstra, Randall J. Wisser, Wenwei Xu, Jianming Yu, Aaron Lorenz

**Affiliations:** ^1^Department of Agronomy and Horticulture, University of Nebraska, Lincoln, NE, United States; ^2^Department of Agronomy, University of Wisconsin, Madison, WI, United States; ^3^Institute for Genomic Diversity, Cornell University, Ithaca, NY, United States; ^4^Department of Crop Sciences, University of Illinois at Urban-Champaign, Urbana, IL, United States; ^5^U.S. Department of Agriculture - Agricultural Research Service Plant, Soil, and Nutrition Research Unit, Cornell University, Ithaca, NY, United States; ^6^Department of Agronomy, Kansas State University, Manhattan, KS, United States; ^7^Department of Agronomy, Iowa State University, Ames, IA, United States; ^8^U.S. Department of Agriculture - Agricultural Research Service Corn Insects and Crop Genetics Research Unit, Iowa State University, Ames, IA, United States; ^9^Iowa Corn Promotion Board, Johnston, IA, United States; ^10^U.S. Department of Agriculture - Agricultural Research Service Plant Genetics Research Unit, University of Missouri, Columbia, MO, United States; ^11^Plant Breeding and Genetics Section, School of Integrative Plant Science, Cornell University, Ithaca, NY, United States; ^12^Plant Science Department, West River Agricultural Center, South Dakota State University, Rapid City, SD, United States; ^13^Department of Agronomy and Plant Genetics, University of Minnesota, St. Paul, MN, United States; ^14^U.S. Department of Agriculture - Agricultural Research Service Plant Science Research Unit, North Carolina State University, Raleigh, NC, United States; ^15^Department of Plant Agriculture, Ridgetown Campus, University of Guelph, Ridgetown, ON, Canada; ^16^U.S. Department of Agriculture - Agricultural Research Service Crop Genetics and Breeding Research Unit, Tifton, GA, United States; ^17^Department of Plant Agriculture, University of Guelph, Guelph, ON, Canada; ^18^Department of Genetics, Development and Cell Biology, Iowa State University, Ames, IA, United States; ^19^Plant Sciences Institute, Iowa State University, Ames, IA, United States; ^20^Department of Plant Science, Penn State University, University Park, PA, United States; ^21^Department of Soil and Crop Sciences, Texas A&M University, College Station, TX, United States; ^22^Plant Pathology and Plant-Microbe Biology Section, School of Integrative Plant Science, Cornell University, Ithaca, NY, United States; ^23^Department of Agronomy, Purdue University, West Lafayette, IN, United States; ^24^Department of Agronomy and Horticulture, University of Nebraska, Lincoln, NE, United States; ^25^Department of Plant and Microbial Biology, University of Minnesota, St. Paul, MN, United States; ^26^Department of Horticulture and Crop Science, The Ohio State University, Columbus, OH, United States; ^27^Department of Plant and Soil Sciences, University of Delaware, Newark, DE, United States; ^28^Texas A&M AgriLife Research, Texas A&M University, Lubbock, TX, United States

**Keywords:** genotype-by-environment interaction (G×E), Genomes to Fields (G2F) initiative, general combining ability (GCA), specific combining ability (SCA), hybrid prediction, genomic prediction

## Abstract

Genomic prediction provides an efficient alternative to conventional phenotypic selection for developing improved cultivars with desirable characteristics. New and improved methods to genomic prediction are continually being developed that attempt to deal with the integration of data types beyond genomic information. Modern automated weather systems offer the opportunity to capture continuous data on a range of environmental parameters at specific field locations. In principle, this information could characterize training and target environments and enhance predictive ability by incorporating weather characteristics as part of the genotype-by-environment (G×E) interaction component in prediction models. We assessed the usefulness of including weather data variables in genomic prediction models using a naïve environmental kinship model across 30 environments comprising the Genomes to Fields (G2F) initiative in 2014 and 2015. Specifically four different prediction scenarios were evaluated (i) tested genotypes in observed environments; (ii) untested genotypes in observed environments; (iii) tested genotypes in unobserved environments; and (iv) untested genotypes in unobserved environments. A set of 1,481 unique hybrids were evaluated for grain yield. Evaluations were conducted using five different models including main effect of environments; general combining ability (GCA) effects of the maternal and paternal parents modeled using the genomic relationship matrix; specific combining ability (SCA) effects between maternal and paternal parents; interactions between genetic (GCA and SCA) effects and environmental effects; and finally interactions between the genetics effects and environmental covariates. Incorporation of the genotype-by-environment interaction term improved predictive ability across all scenarios. However, predictive ability was not improved through inclusion of naive environmental covariates in G×E models. More research should be conducted to link the observed weather conditions with important physiological aspects in plant development to improve predictive ability through the inclusion of weather data.

## Introduction

Genomic prediction and selection have become a powerful tool for plant breeders to develop new improved varieties more quickly and efficiently (Crossa et al., 2017). An initial set of genotyped and phenotyped lines is needed for model calibration. Then, the predictions of untested genotypes can be performed using their marker profiles and the calibrated/developed model. This methodology offers the opportunity to generate maize hybrids by constructing synthetic genotypes from inbred lines whose marker profiles are available (Kadam et al., 2016; Acosta-Pech et al., 2017). Model calibration can also be performed using phenotypic information from hybrids and inbreds previously tested. The creation and prediction of hypothetical hybrids provide a wider inference space to search for superior genotypes with desirable characteristics (high yield, drought tolerance, disease resistance, stable cultivars, etc.) without incurring additional phenotyping costs. Numerous factors affect prediction accuracy, including, among others, the quality of phenotypic and genomic information, the genetic relatedness between training and testing sets, and the trait's genetic architecture (Jarquin et al., 2014).

Generally, breeders are interested in the development of superior cultivars for a wide range of environmental conditions and, therefore, varieties are frequently tested across multiple environments within a breeding program. The identification of superior cultivars is affected by changes in the response patterns of particular genotypes under different environmental stimuli. These changes in the response patterns correspond to changes in the rank performance of the genotypes from one set of environmental conditions to another; the phenomenon is also known as G×E interaction. The understanding of how environmental factors can cause inconsistencies in phenotypic responses of specific genotypes could aid breeders in designing experimental trials in more informed ways. The development and testing of models optimized to predict genotype-by-environment interaction effects is an important area of active research.

Several studies have demonstrated the benefits of modeling the G×E interaction in prediction models (Burgueño et al., 2012; Jarquín et al., 2013; Pérez-Rodríguez et al., 2015; Lado et al., 2016; Basnet et al., 2019). More specifically, Jarquín et al. (2013) and Pérez-Rodríguez et al. (2015) modeled the G×E interaction using an environmental kinship covariance structure that describes the environmental similarities between pairs of environments using weather data. In the absence of weather data this model still allows the inclusion of the G×E interaction by leveraging the interaction between molecular markers and environments instead of the interaction between molecular markers and weather covariates.

Others have extended these implementations allowing the modeling of G×E interaction in a multi-trait context (Malosetti et al., 2016; Montesinos-López et al., 2016). Efforts have also been attempted to integrate genomic selection with crop growth models to better understand the impact of G×E interactions (Heslot et al., 2014; Technow et al., 2014; Onogi et al., 2016; Rincent et al., 2017). Recently, Millet et al. (2019) proposed a model for predicting grain yield as the product of yield components. Such phenotypic dissection might allow the identification of particular components that are less affected by specific environmental conditions (e.g., grain weight). In other cases, a trait (e.g., grain number) might exhibit a strong dependence during key phenological phases such as the amount of radiation intercepted during the vegetative phase, average night temperature, and soil and water potential over the flowering phase. These authors showed that the dissection of grain number via a factorial regression that depends on environmental indices (genotype-specific radiation, soil water potential, and meristem temperature) can substantially enhance the predictive ability compared to current alternatives. However, the computation of some of the needed parameters requires the use of labor intensive or sophisticated phenotyping platforms to record plants' responses and environmental conditions over time, especially during key phenological phases, limiting applications in current field breeding efforts.

The objective of this study was to assess the usefulness of naively modeling environmental covariates (ECs) for the genomic prediction of maize hybrids in different scenarios involving varying levels of available information. Such scenarios include calibrating tested hybrids in observed environments, untested (new) hybrids in observed environments, tested hybrids in unobserved environments, and untested hybrids in unobserved environments. The dataset used here included 30 environments across 21 locations and 2 years (2014 and 2015) collected by the G2F initiative. Models were compared on the basis of prediction accuracies calculated for different cross-validation scenarios. This study will help to elucidate the potential for, and limitations of, the application of environmental data to the genomic prediction for plant breeding when no information about the relationships between the different phenological stages of plant development and the observed weather conditions at these stages are considered. This can help guide future research efforts aimed at optimizing the use of these types of data.

## Materials and Methods

### The G2F Project

The analyzed data were collected by the G2F initiative (www.genomes2fields.org). The initiative started in 2014 with the goal of providing a flexible structure that researchers could participate in and take advantage of to test and evaluate important research questions to help enhance phenotypic predictability (AlKhalifah et al., 2018). This umbrella initiative includes the maize G×E project that aims to annually document and measure genotypes, phenotypes, and environmental data for a collection of diverse maize hybrids at more than 20 locations in North America.

### Genotypes, Environmental Conditions, and Experimental Design

A collection of 1,876 unique maize hybrids was tested in 19 and 24 locations in 2014 and 2015, respectively, representing 17 states in the US and one province in Canada (doi: 10.7946/P2201Q and 10.7946/P24S31, respectively) as part of the G2F Maize G×E project (AlKhalifah et al., 2018). In 2014, hybrids were generated by crossing a total of 380 unique inbred lines to relevant testers. The set of inbred lines included 25 recently expired plant variety protection (EXPVP) lines (Mikel, 2006), 302 were recombinant inbred lines (RILs) derived from biparental populations, and the remaining 53 included lines from diverse origins. For 2014, 23 testers included EXPVP inbred lines LH198, LH195, LH185, and PB80, as well as CG102, which is an inbred line released by the University of Guelph (Lee et al., 2001). As described in Gage et al. (2017), the inbreds used as females in the hybrid combinations for 2014 were classified into eight sets based on their genetic background. A set of 553 unique inbred lines were used in 2015, which included 55 EXPVP lines, 301 lines derived from biparental crosses, and the remaining 197 lines represented diverse lines from various origins and genetic backgrounds. In 2015, the set of testers used also included EXPVP lines PHB47 and PHZ51 for a total of 25 testers. The locations where experiments were grown ranged from latitudes between 30.546919° and 44.994356° and longitudes between −75.465731° and −109.692471°. For more details about specific agronomic practices and growing conditions deployed for each location, please refer to the metadata at dois: 10.7946/P2201Q and 10.7946/P24S31 for 2014 and 2015, respectively. In 2014, sets of ~250 hybrids were grown at each location using two field replications per location for a total of ~500 plots per location. The design was a modified split-plot design with individual sets (based on genetic background) as the whole plot and hybrids as the subplot. In 2015, each location also included ~500 plots but for that year, an incomplete randomized block design was used to expand the number of diverse genotypes included at each location. For each location, this design included 20 blocks. Each location was separated into two replicated blocks with each replicated hybrid planted once in each block. Each block was then further divided into 10 incomplete blocks for placement of check hybrids among the incomplete blocks. From the total 500 plots per location, replications were included in 120 of those plots. Additionally, a set of 10 hybrids (Supplementary Table 1) were planted at each of the two replications at every location in 2014 and 2015. This represented the set of common check hybrids. For both years, hybrids were assigned to specific locations based on expected maturity with the exception of the set of common check hybrids, which were grown across all locations and years, and the locally adapted check hybrids, which were also included in every location, each year, and were specific for each individual location. The experimental design used was the outcome of a large multi-institutional collaboration in which multiple objectives were being addressed. The nature of the randomization and the different type of randomization between years is not expected to affect the answer to the main question of this study, which was the relative benefit of using environmental covariates to increase genomic prediction accuracy of multi-environment trials.

For this study, we used a set of 1,481 of the available 1,876 hybrids [Supplementary Table 2 (2014) and Supplementary Table 3 (2015)] evaluated at least across 30 of the 43 available environments [location-by-year combination (14 in 2014 and 16 in 2015)] for which climatic information was available (see Supplementary Table 3 for details about the setting up of the experiments at each environment). These hybrids were derived by crossing 535 unique inbreds to appropriate testers across both years (25 testers and 510 non-testers) and with 533 inbred lines acting as parent 1 and 25 as parent 2. Overlapping subsets of 206 to 368 hybrids from the 1,481 available hybrids were tested in each of the 30 environments. As previously described (Gage et al., 2017), DNA sequencing data for 232 of the lines used in this overall study were downloaded from the ZeaGBSv2.7 Panzea release (http://www.panzea.org/#!genotypes/cctl). The remaining inbred lines were genotyped with ~900,000 genotyping-by-sequencing (GBS) molecular markers built from the GBS 2.7 and following the protocol described by Elshire et al. (2011). Inbred genotypes were called using the Tassel5-GBS Production Pipeline, and the ZeaGBSv2.7 Production TagsOnPhysicalMap (TOPM) file (AllZeaGBSv2.7_ProdTOPM_20130605.topm.h5, available at panzea.org) was built using data from about 32,000 additional *Zea* samples (Glaubitz et al., 2014). FILLIN40 (Swarts et al., 2014) was used to impute using the available set of maize donor haplotypes with 8k windows (AllZeaGBSv2.7impV5_AnonDonors8k.tar.gz, available at panzea.org). All GBS samples used in this study are listed in Supplementary Tables 2, 4 for the 2014 and 2015 trials, respectively. The complete set of GBS data can be found at doi: 10.7946/P2201Q Genomic information from parental inbred lines was used to derive the hybrid progeny from these crosses via the general and specific combining ability models. The genotypes of the inbred parents were coded based on the number copies of the major allele presents at each locus (0, 1, or 2) in each individual.

### Phenotypes

A total of 8,555 phenotypic records for grain yield (GY) expressed as kg ha^−1^ at 15.5% adjusted grain moisture across 30 environments remained in the analysis after applying quality control measurements. Data points outside the range of the mean within a specific environment plus or minus three standard deviations were discarded as part of the quality control applied for this study. The complete phenotypic data set with information for all hybrids evaluated in 2014 and 2015 can be found at doi: 10.7946/P2V888, folder a._2014_hybrid_phenotypic_data and doi: 10.7946/P24S31, folder a._2015_hybrid_phenotypic_data. A total of 19.3% of all potential combinations between hybrids and environments were evaluated across the 30 locations as part of this study. For 2014 (14 environments) and 2015 (16 environments), 27.7 and 35.6% of all the hybrid-environment combinations were observed, respectively.

### Climatic Information

Each location was equipped with a WatchDogTM Model 2700 (Spectrum Technologies Inc., East-Plainfield, Illinois, 60585, USA) weather station able to capture information on eight environmental covariates (ECs): air temperature (°C), dew point (°C), relative humidity (%), rainfall (mm), wind speed (meters second^−1^), wind direction (degrees), wind gusts (meters second^−1^; largest speed in a 30 min period), and solar radiation (Watts meter^−2^). Nearby stations from the National Weather Service (NWS) Automated Surface Observing Systems (ASOS) were used to verify calibration, identify incorrect data points, and input missing information. Incorrect data points were removed from the datasets and left as missing values. Cleaned and calibrated weather data can be found at doi: 10.7946/P2V888, folder c._2014_weather_data and 10.7946/P24S31, folder c._2015_weather_data for 2014 and 2015, respectively. The calibrated dataset includes observations from the NWS ASOS as well as a “calibrated” column for most elements. In cases where a weather element did not require calibration, the data were simply copied from the regular data column. A more detailed description of the captured environmental information can be found in AlKhalifah et al. (2018) and McFarland et al. (2020).

### Incorporating Climatic Data in Prediction Models

Environmental data were considered in the analysis by including raw climatic data (every data point recorded on an hourly basis by the field weather stations). Setting planting day to zero in all environments offered the opportunity to compare crop performance under similar amounts of recorded weather data. For the 30 environments, there were at least 131 days with (hourly recorded) environmental data available during the growing season. The total number of environmental covariates (ECs) was 25,152, and these were derived from hourly measurements (24) recorded on eight ECs during 131 days (24 × 8 × 131).

### Prediction Models

For modeling the genetic relationships among pairs of hybrids, solely marker information from inbred lines was considered to construct the genomic relationship matrices–[GRMs, VanRaden (2008)] between pairs of hybrids via the general and specific combining ability (GCA and SCA) terms. For the GCA terms, the GRM of parent 1 and parent 2 were built using their corresponding marker profiles (Bernardo, 1994; Technow et al., 2014; Kadam et al., 2016). SCA, which describes the interaction effect of crossing parent 1 with parent 2, was modeled as the Hadamard product of their corresponding GRMs as showed by Acosta-Pech et al. (2017). Finally, the interactions between the GCA and SCA components with environment and ECs were included according to Basnet et al. (2019) and using the reaction norm model proposed by Jarquín et al. (2013). A total of five models were used. The list of models, their components, and corresponding assumptions are provided below:

#### M1. General Combining Ability Model for Main Effects (*G*_*P*1_ + *G*_*P*2_)

This model is the baseline model. Its results are used as the baseline to compare the alternative models (M2–M5). It uses only genomic information from the inbred lines in the analysis via the GCA of the parents (i.e., distinguishing male and female effects) for developing hybrids. This model is composed of an environmental effect *E*_*i*_ ~ *N*(0,*σ^2^_E_*), two genetic scores and an error term. The genetic scores are derived from the main effects of the inbred markers that are involved in the hybrid crosses. Here, for the *j*^th^ hybrid, we denote the corresponding scores for parent 1 and parent 2 as *g*_*P*1*j*_ and *g*_*P*2*j*_, respectively, these being the linear combinations between *p* (*i*=1, 2, …, *m*) markers and their corresponding maker effects. With $g_{P1j}=\sum_{m=1}^{p}x_{P1jm}b_{mP1}$ and $g_{P2j}=\sum_{m=1}^{p}x_{P2jm}b_{mP2};$ *X*_*P*1_ = {*X*_*P*1*j*_}, *X*_*P*2_ = {*X*_*P*2*j*_} are the corresponding inbred marker matrices for parent 1 and parent 2 (Bernardo, 1994; Technow et al., 2014; Kadam et al., 2016); *b*_*mP*1_ and *b*_*mP*2_ are the corresponding effects of the *m*^th^ marker for parent 1 and parent 2 such that $b_{mP1}\overset{iid}{\sim }N(0,\sigma_{bP1}^{2})$ and $b_{mP2}\overset{iid}{\sim }N(0,$, $\sigma_{bP1}^{2}$ and $\sigma_{bP2}^{2}$ act as the associated variance components, and *iid* stands for independent and identically distributed random variables. Using properties of the multivariate normal distribution, we obtained the following linear predictor for modeling the hybrid performance of the *j*^th^ hybrid observed in the *i*^th^ environment via the GCA of the inbreds.

(1) $$\begin{array}{r} y_{ij}=\mu +E_{i}+g_{P1j}+g_{P2j}+e_{ij} \end{array}$$

where ${\text{g}}_{P1}=\left\{g_{P1j}\right\}\sim N\left(0,{\text{G}}_{P1}\sigma_{P1g}^{2}\right)$ and ${\text{g}}_{P2}=\left\{g_{P2j}\right\}\sim N\left(0,{\text{G}}_{P2}\sigma_{P2g}^{2}\right)$ with ${\text{G}}_{P1}=\frac{X_{P1}{X_{P1}}^{\prime }}{p},$ ${\text{G}}_{P2}=\;\frac{X_{P2}{X_{P2}}^{\prime }}{p},$ $\sigma_{P1g}^{2}=p\times \sigma_{bP1}^{2}$ and $\sigma_{P2g}^{2}=p\times \sigma_{bP2}^{2}$ as the correspondent variance components of the parental effects; and $e_{ij}\sim N\left(0,\sigma_{\text{e}}^{2}\right)$ with $\sigma_{\text{e}}^{2}$ being the variance component associated with the residuals. One of the disadvantages of this model is that it does not take into consideration the specific effect of crossing parent 1 with parent 2 (specific combining ability, SCA), but rather the averaged effects of both parents. In this model, a common genomic value is modeled across environments for each hybrid genotype.

#### M2. General Plus Specific Combining Ability Model (Main and Interaction Genetic Effects) (*G*_*P*1_ + *G*_*P*2_ + *G*_*P*1× *P*2_)

This model is an extension of model M1 allowing the inclusion of the SCA interaction effect of crossing a specific pair of parents (Acosta-Pech et al., 2017). SCA was modeled using the cell by cell product of the entries of the co-variance structures from parent 1 and parent 2 such that ${\text{g}}_{P1\times P2}=\left\{g_{P1j\times P2j}\right\}\sim N\left(0,{\text{G}}_{P1\times P2}\sigma_{P1g\times P2g}^{2}\right)$, where **G**_*P*1 × *P*2_ = **G**_*P*1_◦**G**_*P*2_ and $\sigma_{P1g\times P2g}^{2}$ is the variance component associated with this interaction term, and “**°**” denotes the cell by cell product between two matrices, also known as the Hadamard or Schur product.

Combining the assumptions from model M1 with the SCA term, the following linear predictor is obtained.

(2) $$\begin{array}{r} y_{ij}=\mu +E_{i}+g_{P1j}+g_{P2j}+g_{P1j\times P2j}+e_{ij} \end{array}$$

Although this model considers the interaction between the parents, M2 still returns a common genomic value for hybrids across environments, similarly to M1.

#### M3. General Plus Specific Combining Ability Model in Interaction With Environments (*G*_*P*1_ + *G*_*P*2_ + *G*_*P*1×*P*2_ + *G*_*P*1_ × *E* + *G*_*P*2_ × *E* + *G*_*P*1×*P*2_ × *E*)

In an attempt to allow the model to deliver specific responses of the same genotype at different environments, the interaction between the genetic and the environmental components was included. For this, M2 is extended to allow the inclusion of the interaction between the GCA and SCA components with the environments via a reaction norm model (Jarquín et al., 2013).

(3) $$\begin{array}{r} y_{ij}=\mu +E_{i}+g_{P1j}+g_{P2j}+g_{P1j\times P2j}+gE_{P1j}+gE_{P2j}\\ \;+gE_{P1j\times P2j}+e_{ij}, \end{array}$$

where $\begin{array}{l} \text{g}{\text{E}}_{P1}=\left\{gE_{P1j}\right\}\sim N(0,\;({\text{Z}}_{\text{g}P1}{\text{G}}_{P1}{Z^{\prime }}_{\text{g}P1}){}^{\circ}({\text{Z}}_{\text{E}}{Z^{\prime }}_{\text{E}})\sigma_{gEP1}^{2}), \end{array}$ $\begin{array}{l} \text{g}{\text{E}}_{P2}=\left\{gE_{P2j}\right\}\sim N(0,({\text{Z}}_{\text{g}P2}{\text{G}}_{P2}{Z^{\prime }}_{\text{g}P2}){}^{\circ}({\text{Z}}_{\text{E}}{Z^{\prime }}_{\text{E}})\sigma_{gEP2}^{2}), \end{array}$ and $\begin{array}{c} \text{g}{\text{E}}_{P1\times P2}=\left\{gE_{P1j\times P2j}\right\}\sim N(0,({\text{Z}}_{\text{g}P1}{\text{G}}_{P1}{Z^{\prime }}_{\text{g}P1}){}^{\circ}\\ \left({\text{Z}}_{\text{g}P2}{\text{G}}_{P2}{Z^{\prime }}_{\text{g}P2}){}^{\circ}\;({\text{Z}}_{E}{Z^{\prime }}_{E})\sigma_{gEP1\times P2}^{2}\right) \end{array}$ with $\sigma_{gEP1}^{2}$, $\sigma_{gEP2}^{2}$ and $\sigma_{gE\;P1\times P2}^{2}$ as the corresponding variance components of the interaction between the GCA (parent 1 and parent 2) and SCA (*P1* × *P2*) terms with the environments; and **Z_gP1_**, **Z_gP2_** and **Z_E_** are the corresponding incidence matrices to connect **g**_*P*1_, **g**_*P*2_ and **E** = {*E*_*i*_} with **y** = {*y*_*ij*_} (the vector of phenotypic responses). This model still does not allow the interaction of the GCA and/or SCA components with ECs.

#### M4. General Plus Specific Combining Ability Model in Interaction With ECs (*G*_*P*1_ + *G*_*P*2_ + *G*_*P*1×*P*2_ + *G*_*P*1_× *W* + *G*_*P*2_× *W* + *G*_*P*1×*P*2_× *W*)

This model considers the interaction between the GCA and SCA components with the ECs. Here, M3 is modified by replacing the interaction terms with *gw*_*P*1*j*_, *gw*_*P*2*j*_ and *gw*_*P*1*j* × *P*2*j*_ (Jarquín et al., 2013; Basnet et al., 2019).

(4) $$\begin{array}{r} y_{ij}=\mu +E_{i}+g_{P1j}+g_{P2j}+g_{P1j\times P2j}+gw_{P1j}+gw_{P2j}+\\ \;gw_{P1j\;\times \;P2j}+e_{ij} \end{array}$$

Where $\text{g}{\text{w}}_{P2}\sim N(0,\;({\text{Z}}_{\text{g}P1}{\text{G}}_{P1}{Z^{\prime }}_{\text{g}P1}){}^{\circ}\;({\text{Z}}_{\text{E}}\Omega {Z^{\prime }}_{\text{E}})\sigma_{gwP1}^{2}$, $\text{g}{\text{w}}_{P2}\sim N(0,\;({\text{Z}}_{\text{g}P2}{\text{G}}_{P2}{Z^{\prime }}_{\text{g}P2}){}^{\circ}\;({\text{Z}}_{\text{E}}\Omega {Z^{\prime }}_{\text{E}})\sigma_{gwP2}^{2}$ and $\text{g}{\text{w}}_{P1\times P2}\sim N(0,\;({\text{Z}}_{\text{g}P1}{\text{G}}_{P1}{Z^{\prime }}_{\text{g}P1}){}^{\circ}\;({\text{Z}}_{\text{g}P2}{\text{G}}_{P2}{Z^{\prime }}_{\text{g}P2}){}^{\circ}\;({\text{Z}}_{\text{E}}\Omega {Z^{\prime }}_{\text{E}})$ $\sigma_{gwP1\times P2}^{2})$ with $\sigma_{gwP1}^{2}$, $\sigma_{gwP2}^{2}$ and $\sigma_{gwP1\;\times \;P2}^{2}$ being the corresponding variance components for the interaction terms; and $\Omega =\frac{WW^{\prime }}{q}$ as the environmental relationships matrix whose entries describe the environmental similarities between pairs of environments, where *W* is the matrix of *q* ECs.

#### M5. General Plus Specific Combining Ability Model in Interaction With Environments and ECs (*G_*P*1_* + *G_*P*2_* + *G_*P*1×*P*2_* + *G_*P*1_× E* + *G_*P*2_× E* + *G_*P*1×*P*2_× E* + *G_*P*1_× W* + *G_*P*2_× W* + *G_*P*1×*P*2_× W*)

This model is designed to prevent model misspecification due to imperfect environmental data. Here, M3 and M4 are combined into a single model.

(5) $$\begin{array}{r} y_{ij}=\mu +E_{i}+g_{P1j}+g_{P2j}+g_{P1j\;\times \;P2j}+gE_{P1j}+gE_{P2j}\\ \;+gE_{P1j\;\times \;P2j}+gw_{P1j}+gw_{P2j}+gw_{P1j\;\times \;P2j}+e_{ij}, \end{array}$$

where all of the terms are as previously defined.

Supplementary Table 5 summarizes the models used in this study and their corresponding terms. Two of the five models are solely main effect models and the other three also include interactions between the GCA/SCA components and the environments, ECs or both.

### Prediction Schemes

Four different prediction problems relevant to plant breeders were simulated in an attempt to mimic real scenarios that breeders might face while predicting hybrid performance. For the set of genotypes, two states were considered for the training sets: (*i*) tested and (*ii*) untested to indicate whether the genotype from the testing set had been previously tested in another environment (at least once). Similarly, for environments, two states (observed and unobserved) were considered. These indicated whether any portion of the phenotypic information of the target environment is included in the calibration set. The four combinations derived from the combinations of the states of these two factors (genotypes and environments) are: CV2 aiming to predict incomplete field trials mimicking the problem of predicting tested genotypes in observed environments; CV1 attempts to predict newly developed genotypes in observed environments; CV0 predicts already tested lines in unobserved environments; and CV00 predicts untested genotypes in unobserved environments.

### Assessing Predictive Ability

Predictive ability was computed on an environmental basis. For each scenario, the Pearson's correlation coefficient (*r*) between predicted and observed values was calculated for hybrids within the same environment. In order to take the uncertainty around predictions into account and to weight the importance of specific environments based on their corresponding sample sizes, the average predictive ability across environments was computed according to Tiezzi et al. (2017) as:

(6) $$r_{\varphi }=\frac{\sum_{i=1}^{I}\frac{r_{i}}{V\left(r_{i}\right)}}{\sum_{i=1}^{I}\frac{1}{V\left(r_{i}\right)}}$$

with *r*_*i*_ being the Pearson's correlation between predicted and observed values at the *i*^th^ environment, $V\left(r_{i}\right)=\frac{1-r_{i}^{2}}{n_{i}-2}$ is the sampling variance and *n*_*i*_ is the number of observations in the current environment.

## Results

**Figure 1:**
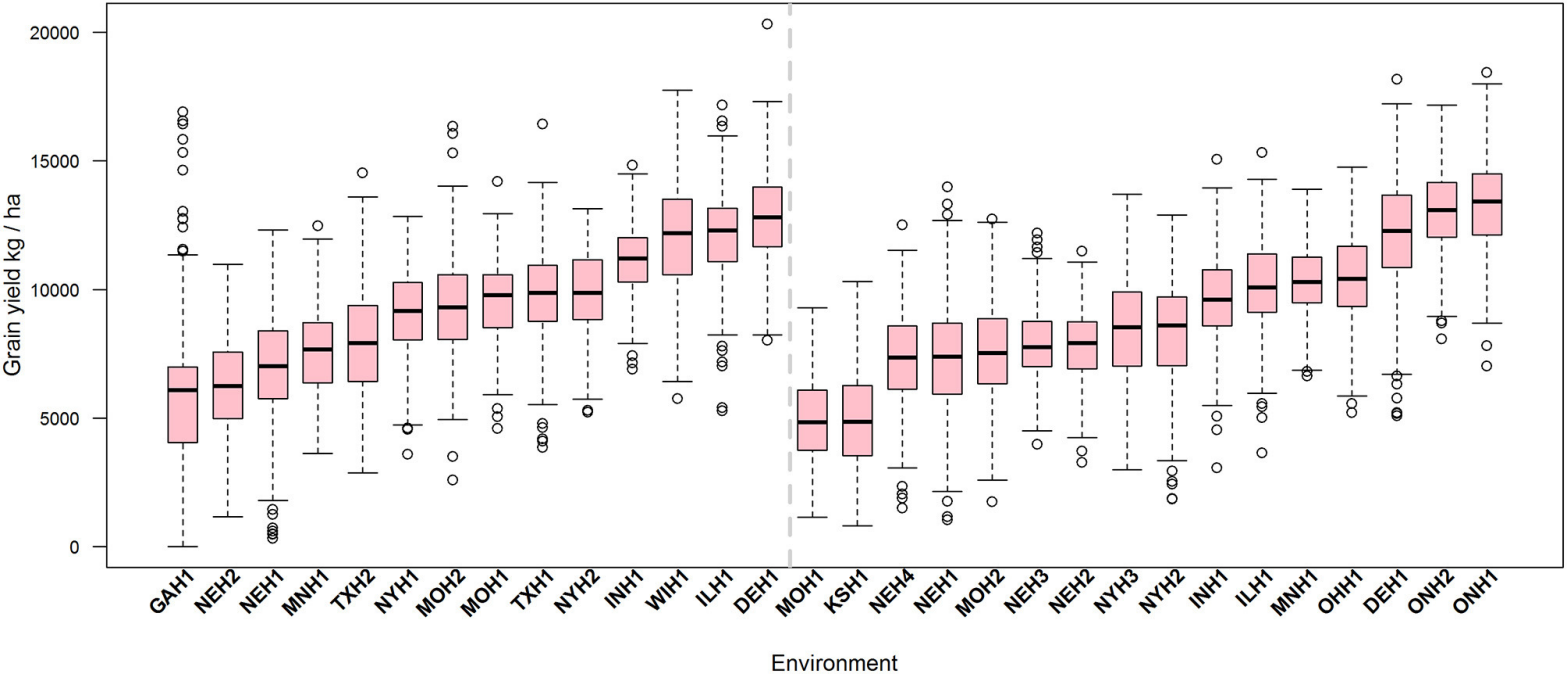
Boxplot of grain yield (kg ha^−1^) of 1,481 unique maize hybrid tested in 30 environments (year-by-location combination; not all of the genotypes were tested in all environments) observed in 2014 (842 genotypes in 14 locations) and in 2015 (928 genotypes in 16 locations) derived from crossing 535 inbreeds to particular testers. Within each environment, those records outside of the interval [μ ± 3 × *SD*] were discarded.

For the total of 8,555 phenotypic (yield) records corresponding to 1,481 hybrids tested in 30 environments that were part of the 2014 and 2015 G2F Maize G×E project, grain yield had a mean of 9065.5 kg ha^−1^ and a standard deviation (SD) of 2979.2 kg ha^−1^ (Supplementary Figure 1). In 2014, 842 unique hybrids (Supplementary Table 6) were tested in 14 environments (mean = 9273.9 kg ha^−1^, SD = 2871.6), whereas in 2015 928 unique hybrids (Supplementary Table 7) were tested in 16 environments (mean = 8904.0 kg ha^−1^, SD = 3033.0). Mean yield ranged between 4910 and 13205.4 kg ha^−1^ across environments showing empirical evidence of environmental differences among trials (Supplementary Table 8). Supplementary Figure 1 depicts the histogram and the empirical distributions for grain yield in 2014, 2015, and both years combined. Figure 1 shows the boxplot of grain yield for those environments observed in 2014 (left panel) and 2015 (right panel), and the environments were ordered based on their medians.

### Analysis of the Variance Components

In general, the environmental term E captured the largest percentage of phenotypic variability 57.2–60.4%) across models (Table 1). In M2, when the SCA term was included the amount of variability explained by GCA was reduced by approximately half. Models that account for interactions of GCA and/or SCA by environment and/or ECs (M3–M5) substantially reduced the amount of variation going into the error. In all cases, the interaction of environment and/or ECs by GCA accounted for more variation than interactions by SCA. When the ECs were used to model the G×E interaction in M4, the amount of variability explained by GCA×W (8.2%) was similar to GCA×E (9.2%) in M3. However, SCA×W (2.9%) explained a larger percentage compared with its counterpart (SCA×E) in M3 (0.1%). The amount of variability explained by the main effects of the molecular markers of parent 1 and parent 2, was similar within models for M1-M5. The same occurred for the G_P1_ × E (4.8%) and G_P2_ × E (4.4%) terms in M3; however, in M5 G_P1_ × E (2.4%) explained at least three times more variability than G_P2_ × E (0.7%). In M4 and M5, G_P2_ × W (5.0% and 4.0%) explained close to twice as much variability as G_P1_ × W (3.2% and 1.9%). The number of unique inbreds acting as parent 1 (533) was at least 21 times more than the number of unique inbreds acting as parent 2 (25) or testers, and 23 of these inbreds acted at least once as parent 1 or parent 2. Perhaps the unbalanced number of unique genotypes acting as parent 1 and parent 2 may have caused these differences in the amount of variability captured by the different components comprising the GCA×E and GCA×W terms (i.e., G_P1_ × E, G_P2_ × E, and G_P1_ × W, G_P2_ × W).

**Table 1:** Percentage of the variability explained by model components of M1–M5.

**Models**	**Main effects**				**Interaction effects**						**Residual**
	**E**	**G_**P1**_**	**G_**P2**_**	**G_**P1 × *P*2**_**	**G_**P1**_ × E**	**G_**P2**_ × E**	**G_**P1 × *P*2**_ × E**	**G_**P1**_ × W**	**G_**P2**_ × W**	**G_**P1 × *P*2**_ × W**	**e**
M1	57.8	6.1	6.2								29.9
M2	60.4	2.9	3.3	4.2							29.9
M3	58.2	2.6	2.8	4.5	4.8	4.4	0.1				22.6
M4	57.0	2.8	2.9	4.3				3.2	5.0	2.9	21.9
M5	57.2	2.7	2.8	4.5	2.4	0.7	0.1	1.9	4.0	2.7	21.1

*E represents the main effect of the environments. G_P1_ and G_P2_ are main effects of inbred markers accounting for paternal/maternal effects (GCA); G_P1×P2_ is the interaction between inbred markers for paternal/maternal effects (SCA); G_P1_ × E and G_P2_ × E are interactions between inbred markers and environments; G_P1_ × W and G_P2_ × W are interactions between inbred markers and ECs; G_P1×P2_ × E is the interaction between SCA effects and environments; G_P1×P2_ × W is the interaction between SCA effects and Environmental Covariates (ECs)*.

### Comparison of Models Under Different Scenarios

#### CV2 Scheme

When attempting to predict tested genotypes in observed environments under the incomplete field trials design (CV2), M1 presented a weighted average correlation of 0.30 (Table 2). Here, the inclusion of the SCA of the parents (M2) enhanced predictive ability to 0.36, 18.6% higher than the baseline model. When the GCA and SCA of the parents were included in interaction with the environments and/or the ECs (M3–M5) the correlation between predicted and observed values was substantially improved to 0.53, 0.50, and 0.53, respectively (66.6 to 76.6% higher than the baseline model). The combination of both interactions gave similar results than the interaction by environment alone. However, when only the interactions between ECs and the GCA/SCA components were included, the weighted average correlation was slightly reduced compared with the other two cases. In this case, we did not observe any clear advantage of including information on ECs (Table 2). A closer look at the trial-by-trial results showed that in 29 out of the 30 environments, M5 outperformed M1 (Supplementary Figure 2).

**Table 2:** Weighted average correlations, 95% and confidence intervals (CI) across 30 environments for five models.

**Model**	**CV2**		**CV1**		**CV0**		**CV00**	
	**Estim**.	**95% CI**	**Estim**.	**95% CI**	**Estim**.	**95% CI**	**Estim**.	**95% CI**
M1	0.30	[0.26, 0.34]	0.25	[0.21, 0.29]	0.29	[0.25, 0.34]	0.10	[0.05, 0.14]
M2	0.36	[0.31, 0.41]	0.31	[0.26, 0.36]	0.35	[0.30, 0.41]	0.20	[0.14, 0.25]
M3	0.53	[0.47, 0.58]	0.46	[0.40, 0.51]	0.45	[0.39, 0.51]	0.27	[0.23, 0.32]
M4	0.50	[0.44, 0.56]	0.43	[0.37, 0.49]	0.26	[0.19, 0.33]	0.17	[0.09, 0.24]
M5	0.53	[0.47, 0.59]	0.46	[0.41, 0.52]	0.47	[0.41, 0.53]	0.29	[0.24, 0.34]

*M1: G_P1_ + G_P2_; M2: G_P1_ + G_P2_ + G_P1×P2_; M3: G_P1_ + G_P2_ + G_P1×P2_ + G_P1_ × E + G_P2_ × E + G_P1×P2_ × E; M4: G_P1_ + G_P2_ + G_P1×P2_ + G_P1_ × W + G_P2_ × W + G_P1×P2_ × W; and M5: G_P1_ + G_P2_ + G_P1×P2_ + G_P1_ × E + G_P2_ × E + G_P1×P2_ × E + G_P1_ × W + G_P2_ × W + G_P1×P2_ × W. G_P1_ and G_P2_ represent the main effects of inbred markers for Parent 1 and Parent 2, respectively. G_P1×P2_ emulates the specific combining ability of crossing Parent 1 and Parent 2. E and W, denote the environment and environmental covariates. The different terms involving _ × E and _ × W represent the interaction between the corresponding marker profiles and the environment or environmental covariates*.

#### CV1

When the objective was to predict new or untested hybrids in already observed environments via other hybrids (CV1) observed in the target and other environments, the average correlation across trials for the reference model M1 was 0.25 (Table 2). As in CV2, the inclusion of the SCA component (M2) enhanced the predictive ability of the reference model to 0.31 (24% higher than M1). When the interactions between inbred markers and environments and/or ECS were included (M3–M5), the reference model was improved to 0.46, 0.43, and 0.46, respectively, which corresponds to improvements between 72 and 84% over M1, respectively. Again, the inclusion of interactions using only weather data did not improve the prediction ability, perhaps because the ECs considered were not appropriate to characterize environments (Table 2) or the environmental signals related with critical stages of the plant development were not clearly identified. Regarding the trial-by-trial results, a similar pattern was observed as for the CV2 scheme, where in 29 of the 30 environments, M5 consistently outperformed M1 (Supplementary Figure 3).

#### CV0 Scheme

Targeting the prediction of already tested hybrids in other environments but not in a new unobserved environment (CV0), the weighted average predictive ability of the M1 was 0.29 (Table 2). When the SCA term was added (M2), the mean correlation was improved to 0.35 (or 21% over M1). The inclusion of the interactions between the GCA and SCA terms with environments (M3) improved the predictive ability to 0.45 (or 55%) compared with M1. In the previous cross-validation schemes, the interactions between GCA and SCA with environmental data (M4) slightly decreased the predictive ability of M3. Here, this type of interaction reduced the predictive ability to 0.26 (or 10%) for M4 compared to M1. Model M5 (0.47) improved the baseline model's result (M1: 0.29) by 62% (Table 2). For individual trials, once again M5, outperformed M1 in 27 out of the 30 environments. M4 outperformed M1 in only 13 out of the 30 environments (Supplementary Figure 4).

#### CV00 Scheme

The prediction of untested hybrids in unobserved environments (CV00) produced an average correlation of 0.10 for the baseline model (M1) (Table 1). As expected, the prediction levels were reduced in this prediction scheme due to (i) the lack of phenotypic information of the hybrids to predict but observed in any other environment, and (ii) the lack of phenotypic records from other hybrids in the target environment. The inclusion of the SCA term via M2 increased the predictive ability to 0.20 (or 100% over M1). M3 and M4 provided substantial improvements when interactions of GCA and SCA components with environments or ECs were included. However, results from M4 model (0.17) were actually worse than those from M3 (0.27). Combining the two types of interactions (M5) lead to a small improvement (0.29) with respect to M3. Thus, the modeling of interaction between ECs and genotypes did not improve predictability. On a trial basis, M5 outperformed M1 in 26 out of the 30 environments (Supplementary Figure 5).

## Discussion

In this study, we showed that genomic prediction models could be improved by considering the interaction of GCA components with environmental factors. These results vary somewhat depending on the cross-validation scheme, but overall, the inclusion of SCA increases predictive ability. However, these improvements are likely due to the fact that both inter- and intra- heterotic group crosses are included in the analyses. It is expected that the benefit of modeling the SCA decreases when analyzing only the inter-heterotic group crosses, which is what applied and commercial breeders do (Melchinger, 1987; Melchinger and Gumber, 1998). In contrast to typical maize breeding programs the percentage of phenotypic variability explained by the SCA term (4.2%) was surprisingly larger than what was explained by the single components of the GCA term, G_P1_ (2.9%) and G_P2_ (3.3%).

For the 1,481 hybrids of this study, there were only 25 unique inbred lines used as parent 2, while 533 acted as parent 1 (of 535 total unique inbred lines). Only PHG35 and C103 inbred lines were used exclusively as parent 2. The use of the SCA term offers the possibility of indirectly capturing the effect of half of the interaction between parent 1 and parent 2 in several crosses that included any of these parents. For a particular hybrid, it is more likely to observe any of the parents involved in the corresponding cross paired with any other inbred than to observe the same inbred combination in the same environment and/or in other environments. Therefore, the sample size for training half of the interaction between parents 1 and 2 involved in the hybrid is increased allowing a better fit. This also facilitates borrowing information within and between environments thus aiding the fit of the models.

Larger improvements were observed when the GCA×E and SCA×E terms were added in M3. The percentage of variability explained by the SCA×E was negligible (0.1%) compared with the 9.2% captured by the GCA×E term. In two (CV0 and CV00) out of the four cross-validation schemes, the average correlation obtained with M5 was slightly superior to the values obtained with the other models (M1–M4). For CV2 and CV1 schemes, M5 returned similar results as M3. In all cross-validation schemes, M2 always increased the average correlation compared to M1. As was mentioned before, the largest portion of improvement was achieved when the interactions between GCA and SCA with the environments was included. Interestingly, when the interactions between these combining ability components and the ECs were included, the obtained average predictive ability was never better than when no ECs were used for characterizing environments. And for CV0, adding these interactions decreased the prediction ability of the baseline model (M1). Combining both types of interactions in M5 did not negatively affect the results of M3.

The lack of improvement in predictive ability through the modeling of environmental covariates contrasts previous findings reported by Jarquín et al. (2013). Jarquín et al. (2013) showed better improvements compared to the baseline model using data on 139 wheat lines tested in 340 environments in which data on 68 ECs based on five phenological phases were collected. That study used similar models to those of this study and improved M3 using M4 between 3 and 12% in CV1 and CV2, respectively; and by around 13% when comparing M5 with M3. Possible reasons why the ECs did not improve predictions in this dataset are (i) small number of environments from a greater range of conditions, (ii) a larger number of more diverse genotypes from different genetic backgrounds, and (iii) the set of environmental covariates included in the model did sufficiently explain similarities among pairs of environments with respect to maize growth and development. These contrasting results from these two studies suggest that modeling ECs using the methods of Jarquín et al. (2013) may benefit predictive ability only when both the number of environments is very large and the range of environmental conditions is narrower compared to those observed in this study. Also, the amount of genetic diversity may have played a role, with greater levels of genetic diversity, as was the case in this study, being harder to leverage for prediction of G×E using ECs than the relatively narrow genetic diversity of advanced breeding lines used in Jarquín et al. (2013). Finally, these different results suggest that ECs should be better defined and selected as they relate to crop growth and development, as the naïve treatment of ECs as was done in this study did not provide any advantage.

The data used in this analysis were collected as part of a large, multi-location and multi-institution initiative and thus the trials were designed to addressed multiple objectives beyond the one that is the subject of this paper. Nevertheless, we feel that the design used did not bias the answer to the main question of this study, which was the determining the benefit of using our “environmental kinship” models to capture G×E effects and thus enhance genomic prediction accuracy for the performance of specific genotypes in specific environments. Our main finding was that these types of models do not benefit prediction accuracy for the reasons we discuss. In fact, we believe that the confounding of genetics with spatial variation would, if anything, lead to an advantage of the environmental kinship model because more related hybrids would be more likely to experience similar environmental effects. Because we did not find this result, we believe that the experimental design used did not bias our results.

Basnet et al. (2019) performed a similar comparison between M4 and M1 for grain yield (wheat) in only three environments. These authors also did not find significant improvements by adding weather information even when dividing the growth cycle into periods of 10 days and then grouping the maximum, minimum, and average temperatures, precipitation, and growing degree-days. The equivalent to models M4 and M1 in their study under the CV2 scheme returned a predictability of 0.64 and 0.62, respectively. It is expected that a better strategy would involve the inclusion of ECs observed during crucial physiological stages of the plant development rather than measurements across the entire life cycle. However, that requires additional yield-related measurements during specific developmental stages during the growing season, which can be labor-intensive. New phenotyping and monitoring technologies are likely to contribute to making this possible.

Recently, Millet et al. (2019) proposed an implementation that outperforms the results of models based on environmental relationships when predicting the yield performance of untested genotypes in unobserved environments. Their cross-validation scheme is similar to our CV00 scheme. These authors modeled grain yield as the product between the estimated individual grain yield and grain number. They used sophisticated phenotyping platforms and conducted repeated measures of the progression of the leaf stage and flowering time for identifying three sensitive indices associated with intercepted radiation, soil water potential (measured with tensiometers), and meristem temperature for dissecting grain number via factorial regressions. These authors showed an improvement in predictive ability of the new implementation with respect to the main effects model of around 55% when predicting maize yield across European environments.

### Predictive Ability Under Different Cross-Validation Schemes

This study considered four different cross-validation schemes mimicking realistic scenarios that breeders face in field research and of interest for improving efficiently in meeting breeding goals. Although the results of these scenarios are commensurable, a direct comparison between them is not trivial since they resemble different prediction problems. As expected, when more information about the genotypes and environments was available, the predictive ability improved. CV2 returned the largest weighted average correlation for all models (M1-M5) compared with the other three schemes. Predicting untested genotypes in unobserved environments (CV00) is the hardest prediction problem. In consequence, it returned the smallest weighted average correlations for all models. The CV1 and CV0 schemes returned similar results except for M4 that reduced its predictive ability by around 45% with respect to M3 in CV0, and by 6% under CV1. In this case, for M1, the lack of information included through unobserved environments in CV0 had a less negative effect on predictive ability compared with the scenario that had unobserved phenotypic record of the hybrids (CV1) in any environment. It should also be considered that for CV0 less information was masked as missing (1/30 ~3.33%) than for CV1 (~20%).

## Conclusions

This study showed the practical advantages of considering G×E interactions for improving predictive ability of tested and untested genotypes in observed and unobserved environments via the GCA and SCA models. Although the inclusion of the G×E interaction improved results considerably with respect to the model that did not include it, no clear advantages in predictive ability were observed when the weather data was naively used for modeling this interaction component. A larger set of environments was likely needed. Previous literature suggests a more elaborate way for including this information beyond an environmental kinship matrix that characterizes the environmental differences between pairs of environments without taking into consideration the information of when or in which stages of the plant development these pieces of information were recorded.

Hence, the obtained results leave open venues to further investigate how the environmental data will be most predictive when included in the models. Possibilities include increasing the environmental sample size, offering the opportunity to consider weather data in a naïve manner, or whether the inclusion of more informed mechanisms to add weather data that are specifically linked to important physiological stages of plant development via environmental indices might allow more accurate estimates even when relatively small sets of environments.

## Data Availability Statement

The original contributions presented in the study are included in the article/Supplementary Materials, further inquiries can be directed to the corresponding author/s.

## Author Contributions

DJ wrote the manuscript and performed the prediction analysis. CR did GBS SNP calling. AL, SK, and EB contributed ideas for analysis and experimental design. Members of the G2F Consortium selected germplasm, designed experiments, phenotype plant materials, compiled, curated phenotypic, and weather data sets. NL recognized the need for, conceived of, and organized the experiment. All authors intellectually contributed to this manuscript.

## Conflict of Interest

The authors declare that the research was conducted in the absence of any commercial or financial relationships that could be construed as a potential conflict of interest.

## Supplementary Material

Supplementary Figure 1 Histogram of grain yield for 2014, 2015 and the combined data (2014 and 2015) and superimposed normal density (blue) for a mean and a standard deviation of 9065.5 and 2979.2 units, respectively. The corresponding densities for the histograms for 2014 (mean = 9273.9, SD = 2871.6) and 2015 (mean = 8904.0, SD = 3033.0) are represented by the black and orange curves, respectively.

Supplementary Figure 2 Predictive ability of models M1–M5 for each one of the observed environments for the cross-validation scenario CV2 (Prediction of tested hybrids in observed environments). M1: G_*P*1_ + G_*P*2_; M2: G_*P*1_ + G_*P*2_ + G_*P*1 × *P*2_; M3: G_*P*1_ + G_*P*2_ + G_*P*1 × *P*2_ + G_*P*1_ × E + G_*P*2_ × E + G_*P*1 × *P*2_ × E; M4: G_*P*1_ + G_*P*2_ + G_*P*1 × *P*2_ + G_*P*1_ × W + G_*P*2_ × W + G_*P*1 × *P*2_ × W; and M5: G_*P*1_ + G_*P*2_ + G_*P*1 × *P*2_ + G_*P*1_ × E + G_*P*2_ × E + G_*P*1 × *P*2_ × E + G_*P*1_ × W + G_*P*2_ × W + G_*P*1 × *P*2_ × W. G_*P*1_ and G_*P*2_ represent the main effects of inbred markers for Parent 1 and Parent 2, respectively. G_*P*1 × *P*2_ emulates the specific combining ability of crossing Parent 1 and Parent 2. E and W denote the environment and environmental covariates. The different terms involving _× E and _× W represent the interaction between the corresponding marker profiles and the environment or environmental covariates. The gray dashed vertical line separates those locations observed in 2014 and 2015, respectively.

Supplementary Figure 3 Predictive ability of models M1–M5 for each one of the observed environments for the cross-validation scenario CV1 (Prediction of untested hybrids in observed environments). M1: G_*P*1_ + G_*P*2_; M2: G_*P*1_ + G_*P*2_ + G_*P*1 × *P*2_; M3: G_*P*1_ + G_*P*2_ + G_*P*1 × *P*2_ + G_*P*1_ × E + G_*P*2_ × E + G_*P*1 × *P*2_ × E; M4: G_*P*1_ + G_*P*2_ + G_*P*1 × *P*2_ + G_*P*1_ × W + G_*P*2_ × W + G_*P*1 × *P*2_ × W; and M5: G_*P*1_ + G_*P*2_ + G_*P*1 × *P*2_ + G_*P*1_ × E + G_*P*2_ × E + G_*P*1 × *P*2_ × E + G_*P*1_ × W + G_*P*2_ × W + G_*P*1 × *P*2_ × W. G_*P*1_ and G_*P*2_ represent the main effects of inbred markers for Parent 1 and Parent 2, respectively. G_*P*1 × *P*2_ emulates the specific combining ability of crossing Parent 1 and Parent 2. E and W denote the environment and environmental covariates. The different terms involving _× E and _× W represent the interaction between the corresponding marker profiles and the environment or environmental covariates. The gray dashed vertical line separates those locations observed in 2014 and 2015, respectively.

Supplementary Figure 4 Predictive ability of models M1-M5 for each one of the observed environments for the cross-validation scenario CV1 (Prediction of tested hybrids in unobserved environments). M1: G_*P*1_ + G_*P*2_; M2: G_*P*1_ + G_*P*2_ + G_*P*1 × *P*2_; M3: G_*P*1_ + G_*P*2_ + G_*P*1 × *P*2_ + G_*P*1_ × E + G_*P*2_ × E + G_*P*1 × *P*2_ × E; M4: G_*P*1_ + G_*P*2_ + G_*P*1 × *P*2_ + G_*P*1_ × W + G_*P*2_ × W + G_*P*1 × *P*2_ × W; and M5: G_*P*1_ + G_*P*2_ + G_*P*1 × *P*2_ + G_*P*1_ × E + G_*P*2_ × E + G_*P*1 × *P*2_ × E + G_*P*1_ × W + G_*P*2_ × W + G_*P*1 × *P*2_ × W. G_*P*1_ and G_*P*2_ represent the main effects of inbred markers for Parent 1 and Parent 2, respectively. G_*P*1 × *P*2_ emulates the specific combining ability of crossing Parent 1 and Parent 2. E and W denote the environment and environmental covariates. The different terms involving _× E and _× W represent the interaction between the corresponding marker profiles and the environment or environmental covariates. The gray dashed vertical line separates those locations observed in 2014 and 2015, respectively.

Supplementary Figure 5 Predictive ability of models M1-M5 for each one of the observed environments for the cross-validation scenario CV00 (Prediction of untested hybrids in unobserved environments). M1: G_*P*1_ + G_*P*2_; M2: G_*P*1_ + G_*P*2_ + G_*P*1 × *P*2_; M3: G_*P*1_ + G_*P*2_ + G_*P*1 × *P*2_ + G_*P*1_ × E + G_*P*2_ × E + G_*P*1 × *P*2_ × E; M4: G_*P*1_ + G_*P*2_ + G_*P*1 × *P*2_ + G_*P*1_ × W + G_*P*2_ × W + G_*P*1 × *P*2_ × W; and M5: G_*P*1_ + G_*P*2_ + G_*P*1 × *P*2_ + G_*P*1_ × E + G_*P*2_ × E + G_*P*1 × *P*2_ × E + G_*P*1_ × W + G_*P*2_ × W + G_*P*1 × *P*2_ × W. G_*P*1_ and G_*P*2_ represent the main effects of inbred markers for Parent 1 and Parent 2, respectively. G_*P*1 × *P*2_ emulates the specific combining ability of crossing Parent 1 and Parent 2. E and W denote the environment and environmental covariates. The different terms involving _× E and _× W represent the interaction between the corresponding marker profiles and the environment or environmental covariates. The gray dashed vertical line separates those locations observed in 2014 and 2015, respectively.
