# Supplementary material for: Utility of Climatic Information via Combining Ability Models to Improve Genomic Prediction for Yield Within the Genomes to Fields Maize Project
Source: Front Genet. 2021 Mar 8;11:592769. doi: 10.3389/fgene.2020.592769 (PMC7982677; doi:10.3389/fgene.2020.592769)
Supplement: Supplementary Figure 1 — Histogram of grain yield for 2014, 2015 and the combined data (2014 and 2015) and superimposed normal density (blue) for a mean and a standard deviation of 9065.5 and 2979.2 units, respectively. The corresponding densities for the histograms for 2014 (mean = 9273.9, SD = 2871.6) and 2015 (mean = 8904.0, SD = 3033.0) are represented by the black and orange curves, respectively. [file Data_Sheet_1.docx]

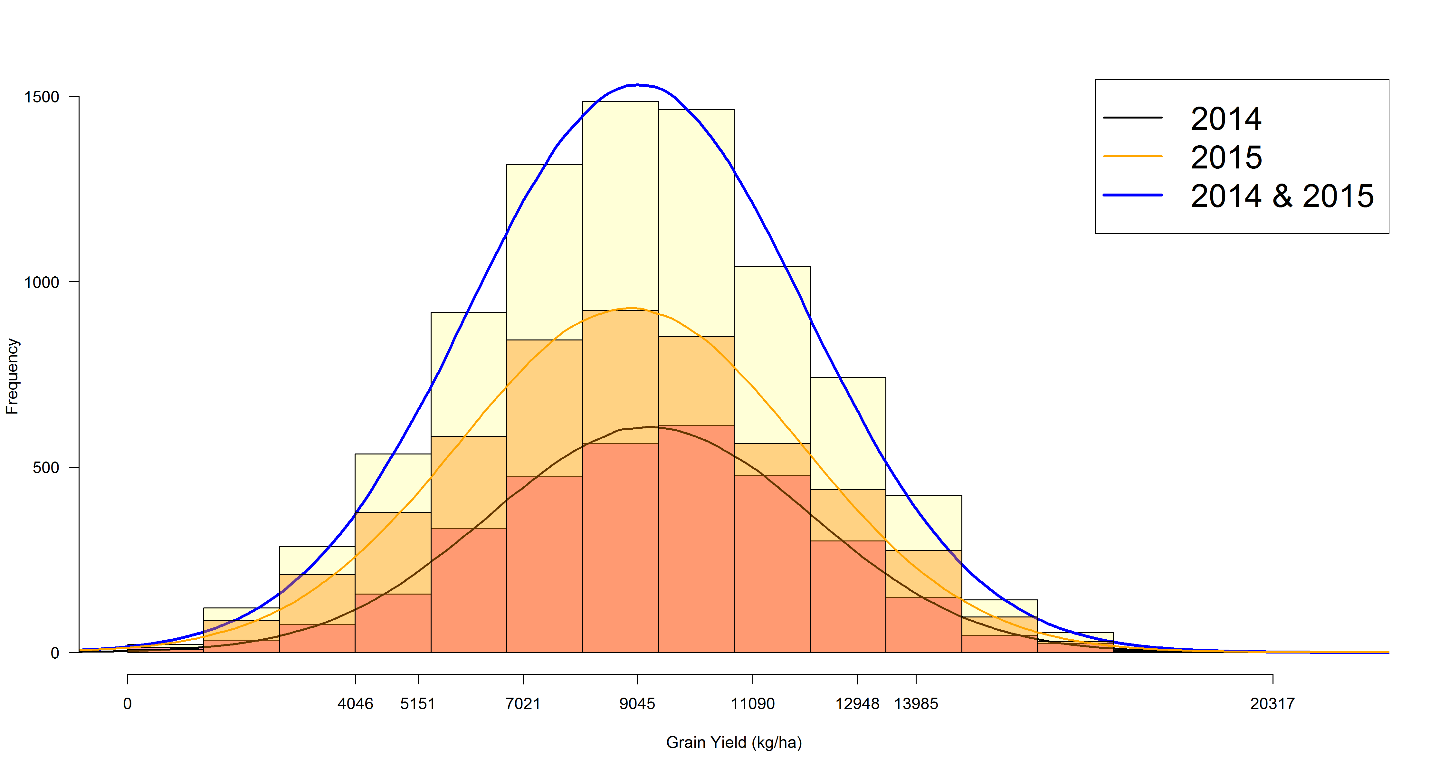


Supplemental Figure S1. Histogram of grain yield for 2014, 2015 and the combined data (2014 & 2015) and superimposed normal density (blue) for a mean and a standard deviation of 9065.5 and 2979.2 units, respectively. The corresponding densities for the histograms for 2014 (mean=9273.9, SD=2871.6) and 2015 (mean=8904.0, SD=3033.0) are represented by the black and orange curves, respectively.


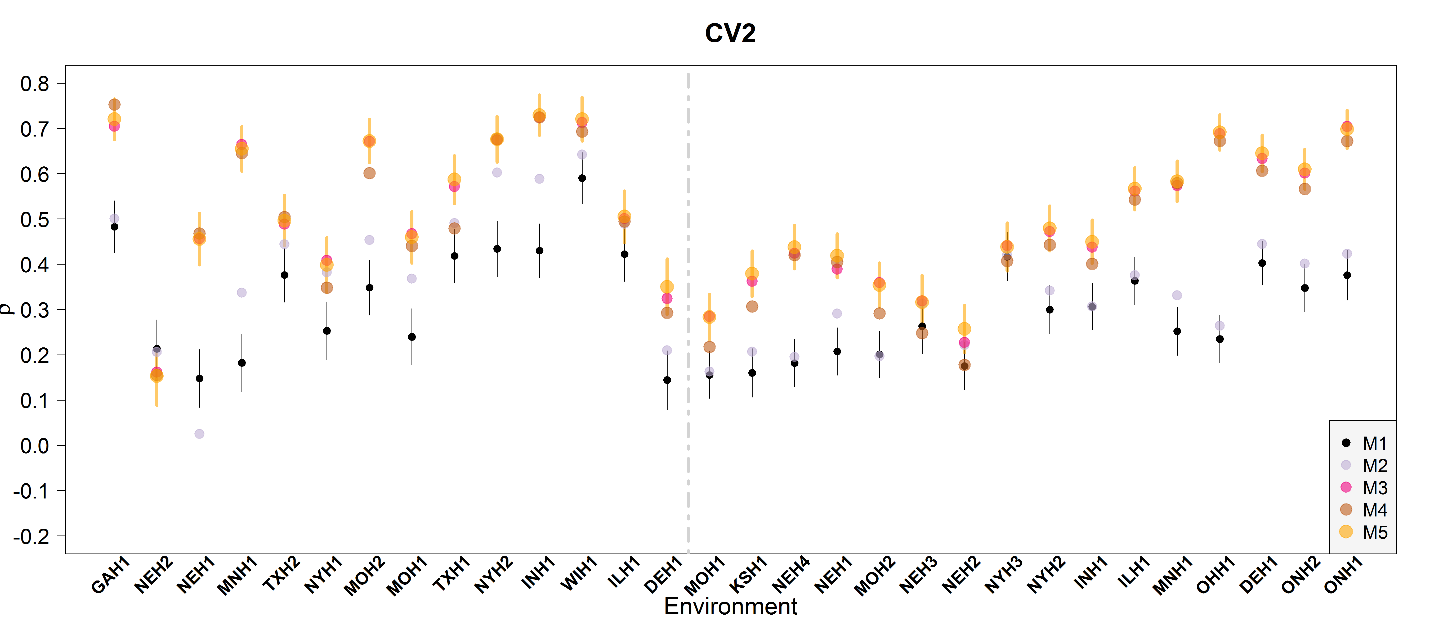


Supplemental Figure S2. Predictive ability of models M1-M5 for each one of the observed environments for the cross-validation scenario CV2 (Prediction of tested hybrids in observed environments). M1: G_P1_ + G_P2_; M2: G_P1_ + G_P2_ + G_P1 × P2_; M3: G_P1_ + G_P2_ + G_P1 × P2_ + G_P1_ × E + G_P2_ × E + G_P1 × P2_ × E; M4: G_P1_ + G_P2_ + G_P1 × P2_ + G_P1_ × W + G_P2_ × W + G_P1 × P2_ × W; and M5: G_P1_ + G_P2_ + G_P1 × P2_ + G_P1_ × E + G_P2_ × E + G_P1 × P2_ × E + G_P1_ × W + G_P2_ × W + G_P1 × P2_ × W. G_P1_ and G_P2_ represent the main effects of inbred markers for Parent 1 and Parent 2, respectively. G_P1 × P2_ emulates the specific combining ability of crossing Parent 1 and Parent 2. E and W denote the environment and environmental covariates. The different terms involving _× E and _× W represent the interaction between the corresponding marker profiles and the environment or environmental covariates. The gray dashed vertical line separates those locations observed in 2014 and 2015, respectively.


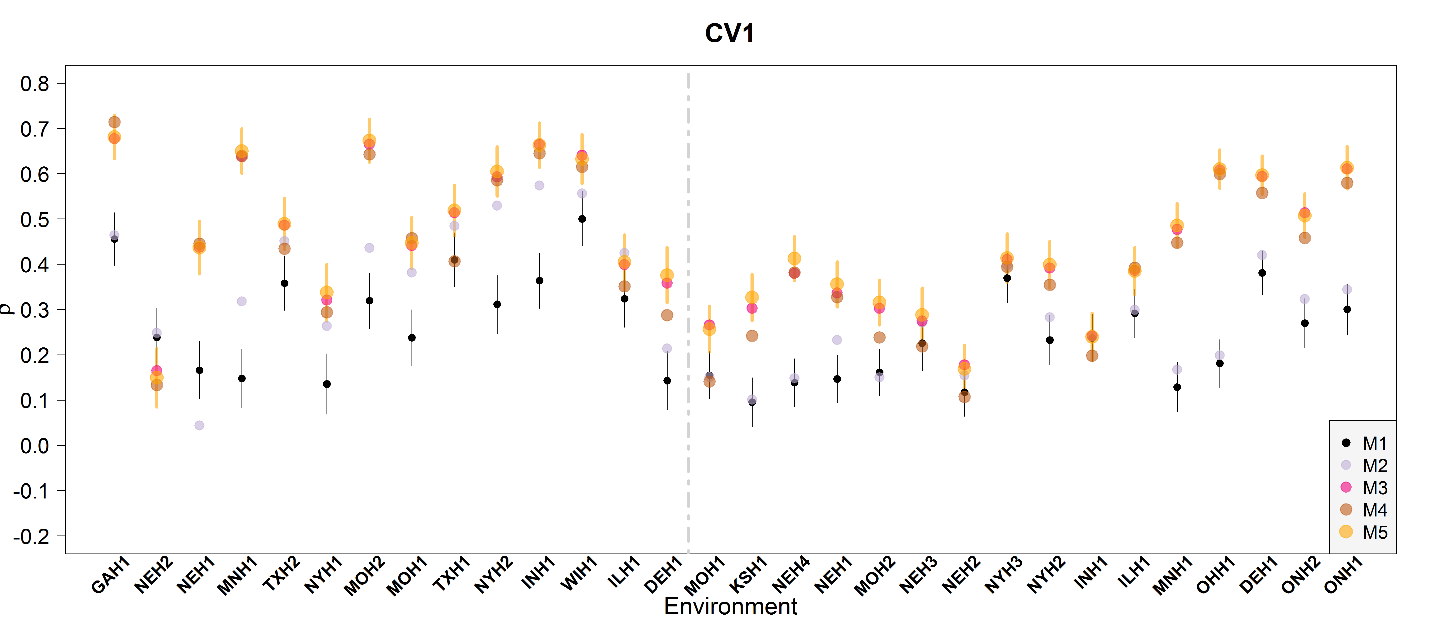


Supplemental Figure S3. Predictive ability of models M1-M5 for each one of the observed environments for the cross-validation scenario CV1 (Prediction of untested hybrids in observed environments). M1: G_P1_ + G_P2_; M2: G_P1_ + G_P2_ + G_P1 × P2_; M3: G_P1_ + G_P2_ + G_P1 × P2_ + G_P1_ × E + G_P2_ × E + G_P1 × P2_ × E; M4: G_P1_ + G_P2_ + G_P1 × P2_ + G_P1_ × W + G_P2_ × W + G_P1 × P2_ × W; and M5: G_P1_ + G_P2_ + G_P1 × P2_ + G_P1_ × E + G_P2_ × E + G_P1 × P2_ × E + G_P1_ × W + G_P2_ × W + G_P1 × P2_ × W. G_P1_ and G_P2_ represent the main effects of inbred markers for Parent 1 and Parent 2, respectively. G_P1 × P2_ emulates the specific combining ability of crossing Parent 1 and Parent 2. E and W denote the environment and environmental covariates. The different terms involving _× E and _× W represent the interaction between the corresponding marker profiles and the environment or environmental covariates. The gray dashed vertical line separates those locations observed in 2014 and 2015, respectively.


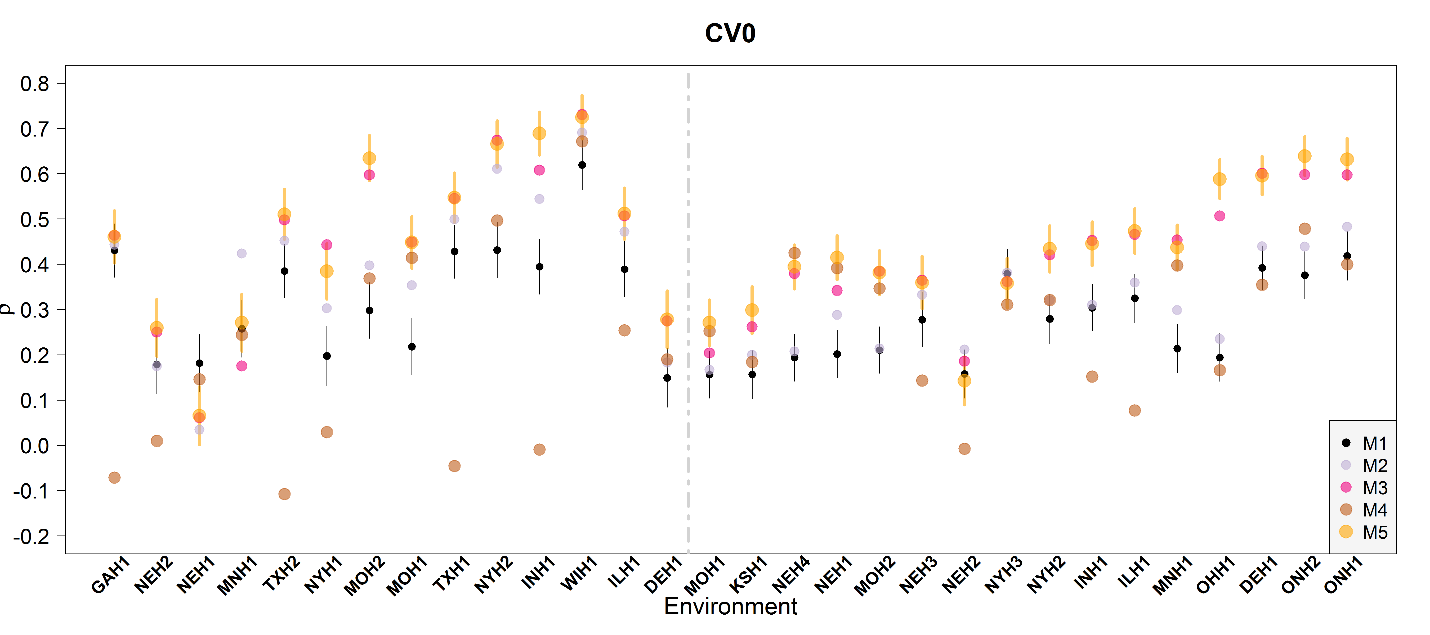


Supplemental Figure S4. Predictive ability of models M1-M5 for each one of the observed environments for the cross-validation scenario CV1 (Prediction of tested hybrids in unobserved environments). M1: G_P1_ + G_P2_; M2: G_P1_ + G_P2_ + G_P1 × P2_; M3: G_P1_ + G_P2_ + G_P1 × P2_ + G_P1_ × E + G_P2_ × E + G_P1 × P2_ × E; M4: G_P1_ + G_P2_ + G_P1 × P2_ + G_P1_ × W + G_P2_ × W + G_P1 × P2_ × W; and M5: G_P1_ + G_P2_ + G_P1 × P2_ + G_P1_ × E + G_P2_ × E + G_P1 × P2_ × E + G_P1_ × W + G_P2_ × W + G_P1 × P2_ × W. G_P1_ and G_P2_ represent the main effects of inbred markers for Parent 1 and Parent 2, respectively. G_P1 × P2_ emulates the specific combining ability of crossing Parent 1 and Parent 2. E and W denote the environment and environmental covariates. The different terms involving _× E and _× W represent the interaction between the corresponding marker profiles and the environment or environmental covariates. The gray dashed vertical line separates those locations observed in 2014 and 2015, respectively.


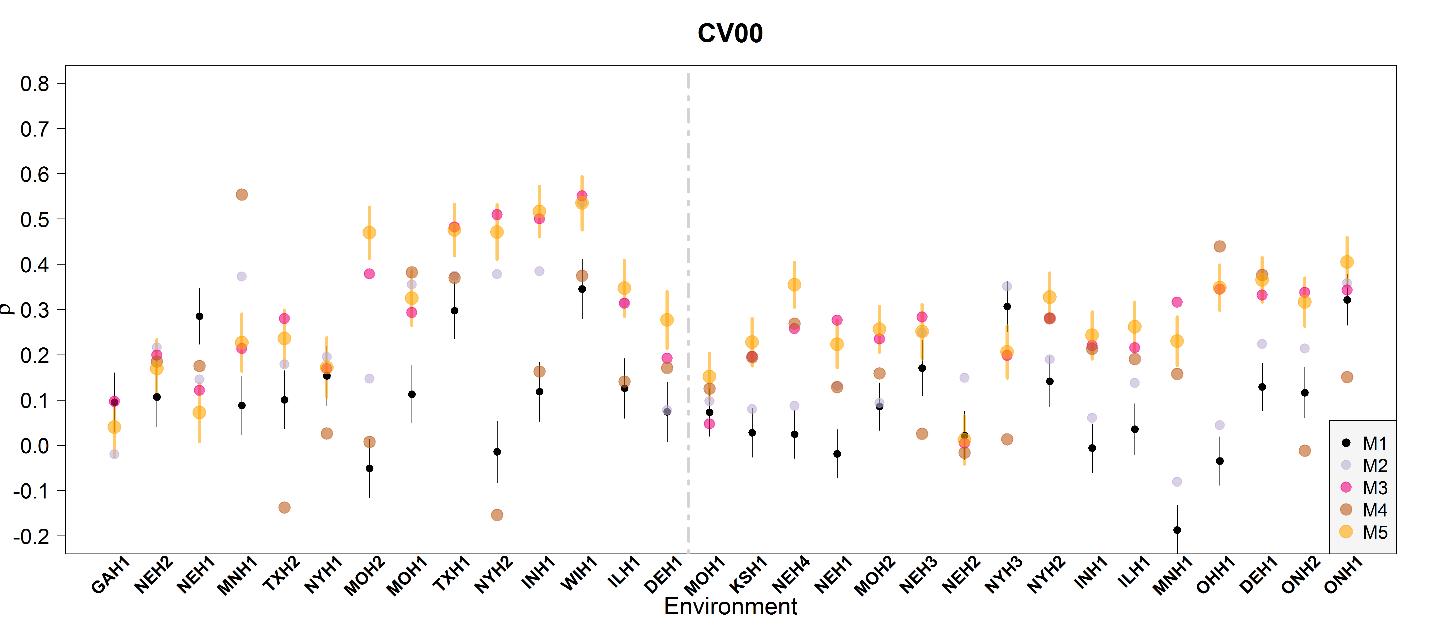


Supplemental Figure S5. Predictive ability of models M1-M5 for each one of the observed environments for the cross-validation scenario CV00 (Prediction of untested hybrids in unobserved environments). M1: G_P1_ + G_P2_; M2: G_P1_ + G_P2_ + G_P1 × P2_; M3: G_P1_ + G_P2_ + G_P1 × P2_ + G_P1_ × E + G_P2_ × E + G_P1 × P2_ × E; M4: G_P1_ + G_P2_ + G_P1 × P2_ + G_P1_ × W + G_P2_ × W + G_P1 × P2_ × W; and M5: G_P1_ + G_P2_ + G_P1 × P2_ + G_P1_ × E + G_P2_ × E + G_P1 × P2_ × E + G_P1_ × W + G_P2_ × W + G_P1 × P2_ × W. G_P1_ and G_P2_ represent the main effects of inbred markers for Parent 1 and Parent 2, respectively. G_P1 × P2_ emulates the specific combining ability of crossing Parent 1 and Parent 2. E and W denote the environment and environmental covariates. The different terms involving _× E and _× W represent the interaction between the corresponding marker profiles and the environment or environmental covariates. The gray dashed vertical line separates those locations observed in 2014 and 2015, respectively.
